# Editorial: Motivation seen through the kaleidoscope of multi-disciplinarity and multi-scales: towards the emergence of new paradigms and perspectives favored by crossed looks

**DOI:** 10.3389/fnbeh.2025.1728916

**Published:** 2025-11-20

**Authors:** Bénédicte Terrier, Jackson Cioni Bittencourt, Sandrine Parrot

**Affiliations:** 1 Université Claude Bernard Lyon 1, INSERM, CNRS, Centre de Recherche en Neurosciences de Lyon CRNL U1028 UMR5292, Bron, France; 2 Laboratory of Chemical Neuroanatomy, Department of Anatomy, Institute of Biomedical Sciences, University of Sao Paulo, Sao Paulo, Brazil

**Keywords:** motivation, transdisciplinary, perspective, integrative theories, personalized interventions

Motivation is a powerful driving force in the dynamics of animal and human behavior. It is based on complex brain processes that are built up and developed over time throughout life. Motivational processes have strong evolutionary and ecological foundations, as they are linked to the survival of the species, encompassing caring behavior, learning, commitment to change, and social interactions, among others. Although it is one of the most widely addressed topics in behavioral research, many questions remain unanswered because motivation has multiple facets. In this context, the bibliometric analysis by Helou and Bittencourt maps the landscape of motivation research, notably driving reward and addiction, underlined the interconnections between neuroscience, psychology, and behavioral sciences linking animal models and studies carried out on human populations. The present editorial of the Research Topic gathering also includes seven research articles, which are representative of the progress in the field of motivation, and have been classically divided into the approaches carried out on animals on one hand, and those related to human motivation on the other hand.

Motivation in animals can currently be appreciated through the interaction of factors such as genetics, life experiences, and developmental stages, internal states, environmental context, and cost dynamics, as well as sex differences and prior reinforcement patterns. Four studies explore motivation from a more individualized and context-sensitive perspective. Mejaes et al. studied the Parkinson's-related gene SYNJ1, suspected to be involved in the lack of motivation in Parkinson's patients. The authors explained how a genetic molecular risk factor, associated with significant sex-specific variations, can influence motivational behaviors via dopamine signaling (Mejaes et al.). In a developmental study, Zeng et al. investigated how adolescent rats, challenged in a rodent gambling task with win-paired cues designed to reproduce human gambling environments, performed as compared to adult rats. Comparing adolescent and adult responses shows that risk-taking isn't always driven by contextual cues in the same way and the findings challenge the idea that risky choices underline on the same neuronal circuits. Using a task inspired by natural foraging, Wittek et al. introduced a semi-naturalistic and ecological grounded way to measure motivation in pigeons. They showed by measuring delay, probability, and informational reward value that those animals don't always follow the strategies currently regarded as optimal, suggesting that the decisions taken in a natural are guided by a mix of individual preference and situational constraints. In a last study with a therapeutic aim in combined substance abuse encountered in humans, Robison et al. examined, using a reinforcement demand model, how male rats self-administer ethanol and nicotine over sessions and they showed that the motivation varies between and within subjects, especially when faced with different types of reinforcement.

When considering the articles conducted on humans, we can stress on the fact that the three studies emphasize both interdisciplinary integrations, including neuroscience, psychology, and education, and the use of innovative methods, such as narrative inquiry, to understand how intrinsic self-fulfillment and context-specific climates drive behavior and professional commitment. Girard and de Guise present a study which compared pupils of pre-service teachers who received the “*learning how to motivate*” training against a control group, to address the need for explicit motivational strategy instruction during physical education classes. It concluded that the training effectively helped trained teachers apply theory, resulting in trained pupils perceiving significantly higher empowering motivational climate dimensions (autonomy support, relatedness support) at the internship's end compared to control pupils, whose perceptions decreased and whose adopt more of performance-approach goals. In another study, in the current context of education shortage, Zhou et al. explored dynamic career motivation changes in pre-service Chinese as a second language (CSL) teachers. The narrative inquiry conducted concluded that intrinsic motivation is the primary driver for career choices and narratives showed that positive practicum experiences and recognition of intercultural motivations enhance commitment, while rigid systems and lack of guidance can diminish self-efficacy and lead to abandoning the career as a CSL teacher. Finally, the study of Cai and Yang investigated how online social media influences as a “double-edged sword” the motivation and regulation of Chinese interpreting learners in self-regulated learning settings, through online social media. It boosts motivation via emotional support and resources, e.g., “speech bank,” but simultaneously acts as a deterrent due to distraction, misinformation, and anxiety from peer comparison, bridging motivation to action in the digital environment.

To conclude, while Helou and Bittencourt also stressed out the motivation field's transformation from basic animal studies to complex human research integrating neurobiological and social factors, they also identified a recent shift toward studying motivational deficits, like apathy and anhedonia, emphasizing the interdisciplinary importance of dopamine and reward systems. However, taken together, all the eight recent studies gathered in the Research Topic bring new stones in four major long-term trends on motivation: (i) the ecological/modern contexts help redefine the concept of motivation (Wittek et al.; Zhou et al.; Cai and Yang); (ii) the priority in the field is given to research presenting novel empirical data and integrative models (Robison et al.; Zeng et al.; Girard and de Guise; Mejaes et al.); (iii) encouraging a cross-disciplinary dialogue can open explored perspectives and foster transdisciplinary discussion (Helou and Bittencourt; Wittek et al.; Cai and Yang); (iv) a key goal is to connect disciplines for further new intervention strategies (Robison et al.; Zeng et al.; Girard and de Guise; Mejaes et al.).

